# Supplementary material for: Spatially Enriched Paralog Rearrangements Argue Functionally Diverse Ribosomes Arise during Cold Acclimation in Arabidopsis
Source: Int J Mol Sci. 2021 Jun 7;22(11):6160. doi: 10.3390/ijms22116160 (PMC8201131; doi:10.3390/ijms22116160)
Supplement: Supplementary file 1 [file ijms-22-06160-s001.zip › legend.pdf]

Figure S1. Hydroponic system used to grow plants, Figure S2. Example of the distribution testing procedure, Figure S3. Significantly changed ribosome-associated proteins (RAP) and ribosomal protein (RP) probe hybridization during cold acclimation, Figure S4. Original exemplary ribosomal sedimentation profiles from plant root tissue, Figure S5. Relative abundances and presence/absence calls of candidate ribosomal proteins during cold acclimation of *Arabidopsis thaliana* roots across independent experiments and genotypes, Figure S6. Ribosomal regions significantly enriched in cold-induced changes of transcript or protein molecular species in *Arabidopsis thaliana* root systems, Figure S7. Unique peptide abundances belonging to the paralog uL30-AtRPL7A that appeared only during cold acclimation in shoot tissue in our experiments, Figure S8. Putative binding site of Rei1 in its polypeptide exit tunnel inserted mode, Figure S9. Detected molecular species belonging to RP coding genes among transcriptomic and proteomic cold acclimation experiments in *Arabidopsis thaliana* roots, Table S1A. Successive dry weight measurements of Arabidopsis thaliana seedlings reared until the stage 1.10 and shifted for a 7 day period of cold acclimation, Table S1B. Calibration curve used in Table S1A,C. Control of weighing fluctuations during the days of the experiment using fixed metal objects as constants, Table S2A. Significantly changed probe hybridization of *Arabidopsis thaliana* Col-0 wild-type cytosolic ribosomal protein (RPs) transcripts after 1 and 7 days of cold acclimation, Table S2B. Significantly changed probe hybridization of *Arabidopsis thaliana* Col-0 wild-type ribosome-associated protein (RAPs) transcripts after 1 and 7 days of cold acclimation, Table S2C. Clusters of expression patters among significantly changed RPs during cold acclimation, Table S3.Legend. Normalized LFQ abundances of 40S and 60S RPs measured across 40S, 60S, 80S, to polysome fractions of *Arabidopsis thaliana* Col-0 wild type and two *reil1 reil2* mutants' roots, Table S3. Master table. The MaxQuant identifier of proteins comprising gene code and variant number are listed in the first column, Table S4. RP paralog accumulation in polysomes compared to the non-translating LSU subunit fractions across multiple sample types, Table S5. Mean ratios of 20 °C/10 °C candidate ribosomal protein abundances during cold acclimation in *Arabidopsis thaliana* roots across independent experiments and genotypes in active translating polysomes, Table S6. Binary input for the local enrichment test of transcriptome or proteome significances as detailed in the GitHub repository COSNet$_i$, Table S7. Gene sequence alignment metadata. Table S8. Ribosome biogenesis factors (RBF) following expression patterns associated with Figure S3.
