# Supplementary figures and images for: Spatially Enriched Paralog Rearrangements Argue Functionally Diverse Ribosomes Arise during Cold Acclimation in Arabidopsis
Source: Int J Mol Sci. 2021 Jun 7;22(11):6160. doi: 10.3390/ijms22116160 (PMC8201131; doi:10.3390/ijms22116160)

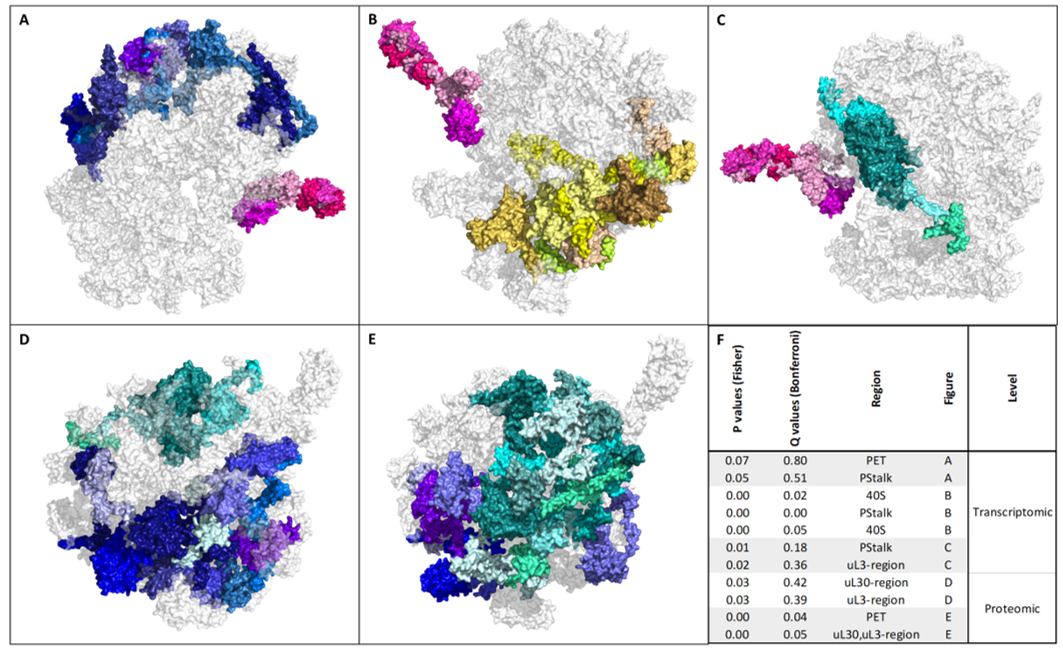

Supplement: Supplementary file 1 [file ijms-22-06160-s001.zip › Figure S6.tif]

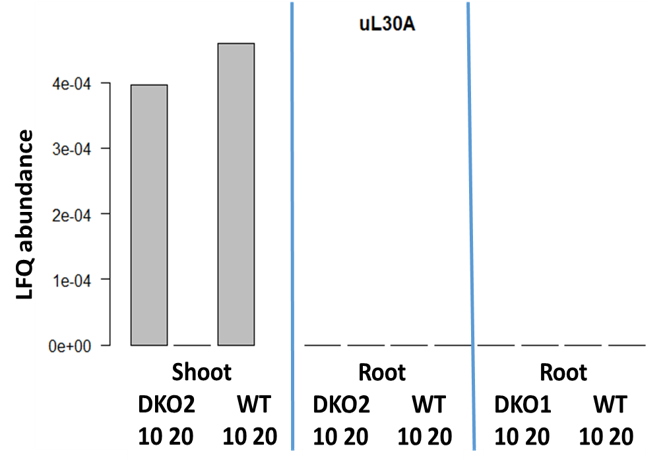

Supplement: Supplementary file 1 [file ijms-22-06160-s001.zip › Figure S7.tif]

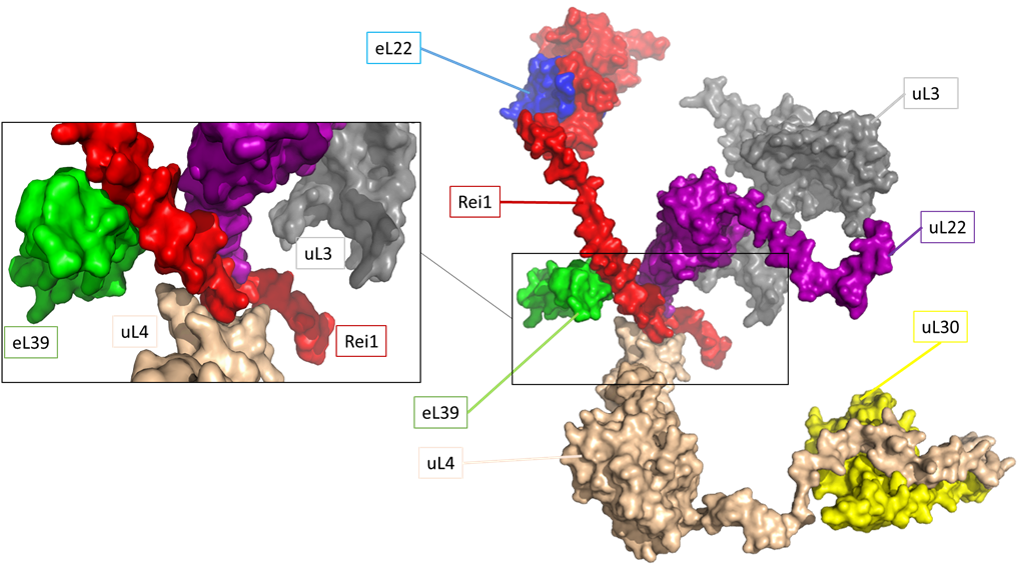

Supplement: Supplementary file 1 [file ijms-22-06160-s001.zip › Figure S8.tif]

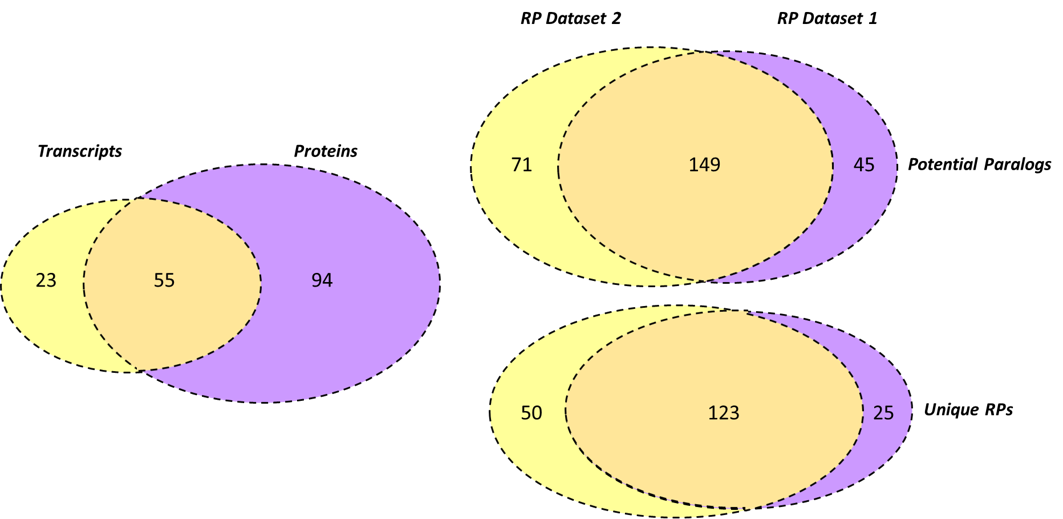

Supplement: Supplementary file 1 [file ijms-22-06160-s001.zip › Figure S9.tif]

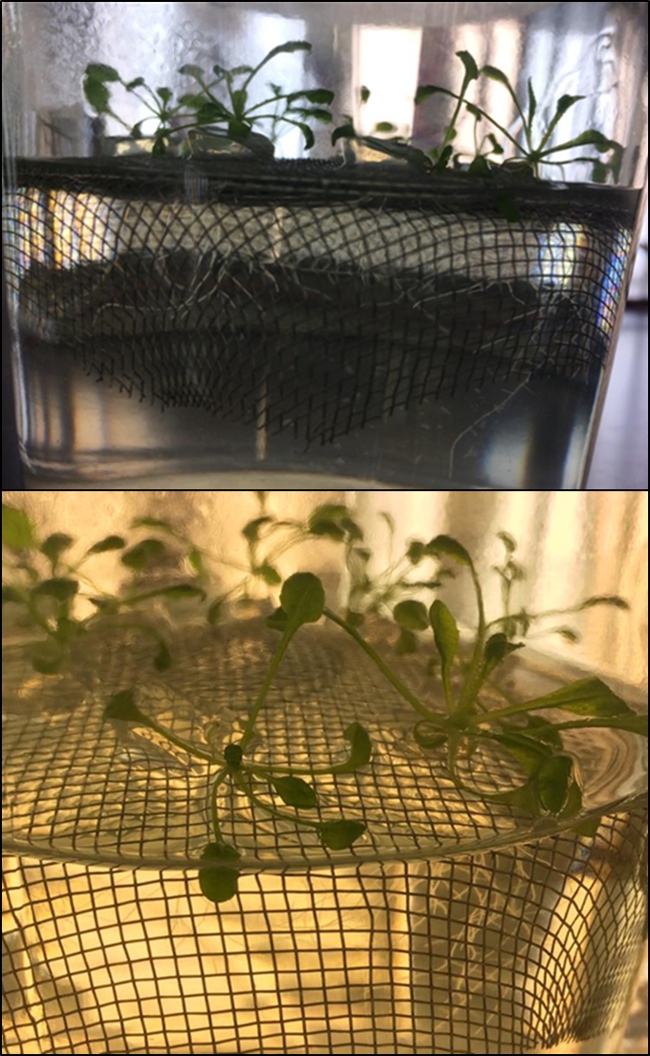

Supplement: Supplementary file 1 [file ijms-22-06160-s001.zip › Figure S1.tif]

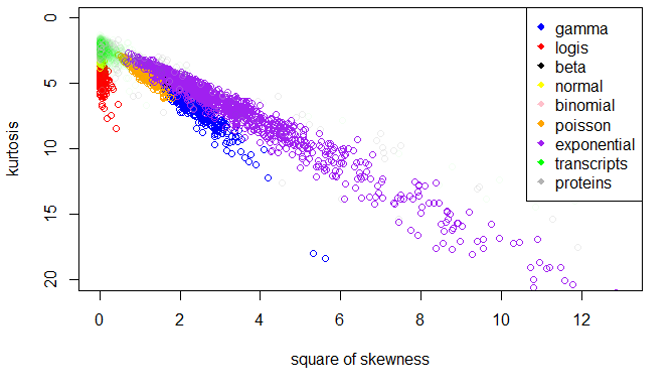

Supplement: Supplementary file 1 [file ijms-22-06160-s001.zip › Figure S2.tif]

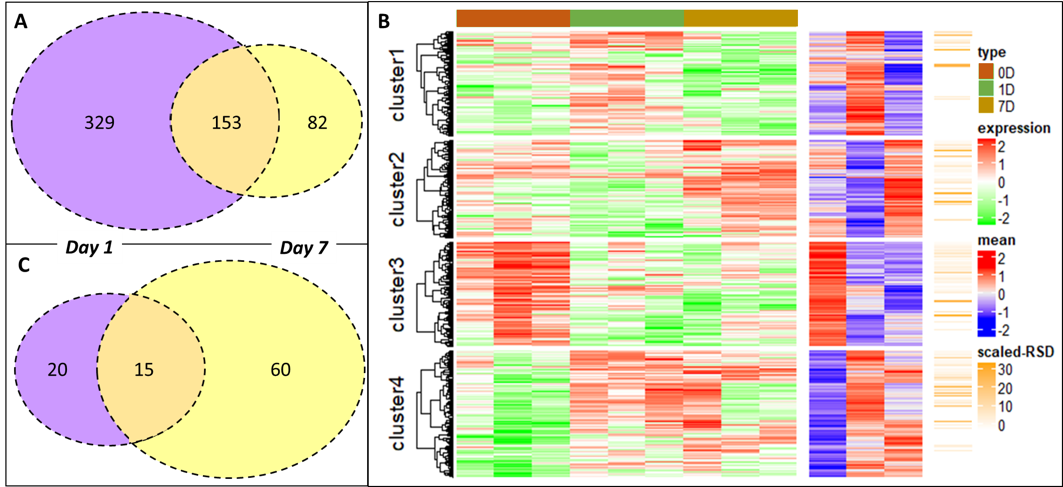

Supplement: Supplementary file 1 [file ijms-22-06160-s001.zip › Figure S3.tif]

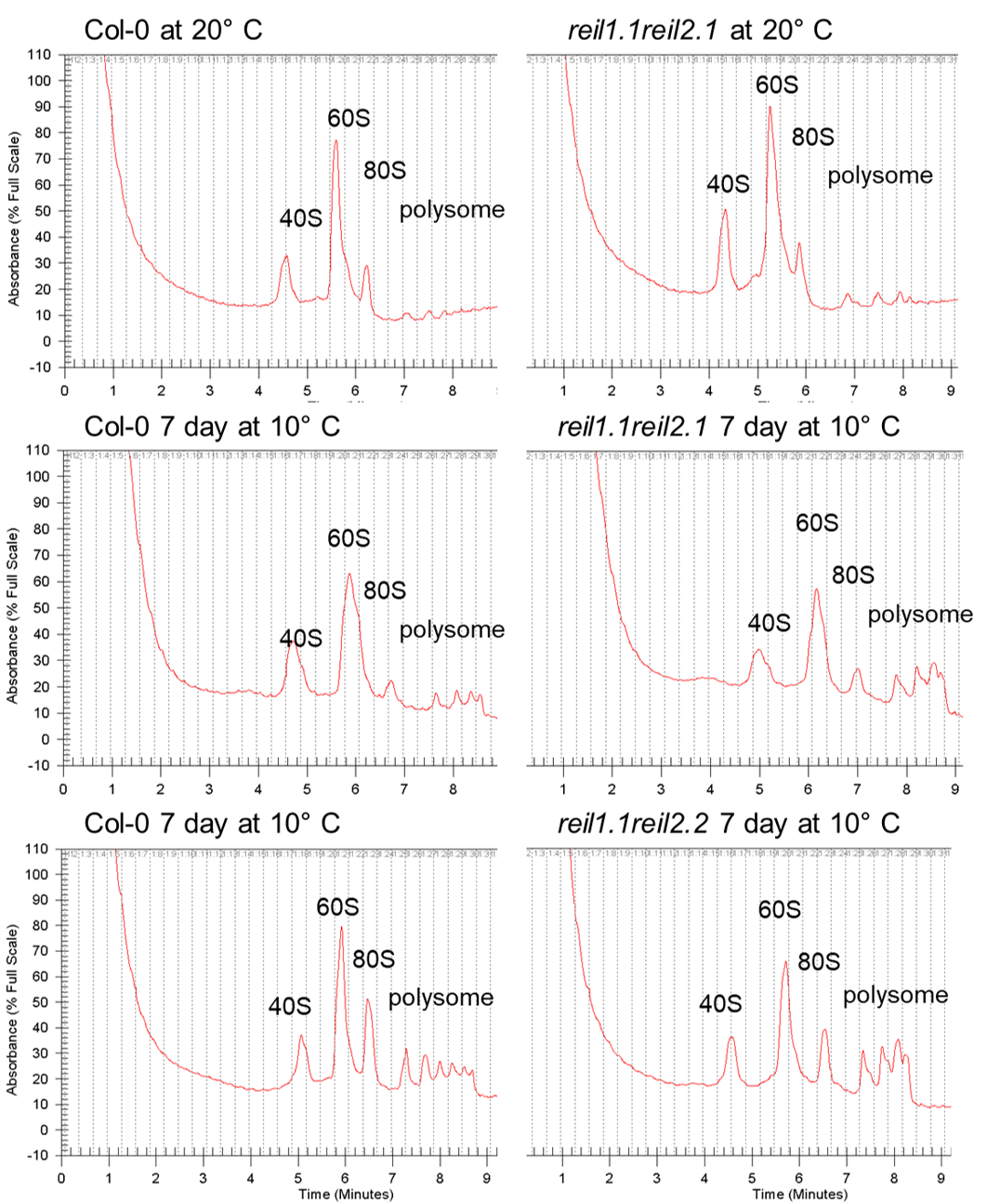

Supplement: Supplementary file 1 [file ijms-22-06160-s001.zip › Figure S4.tif]

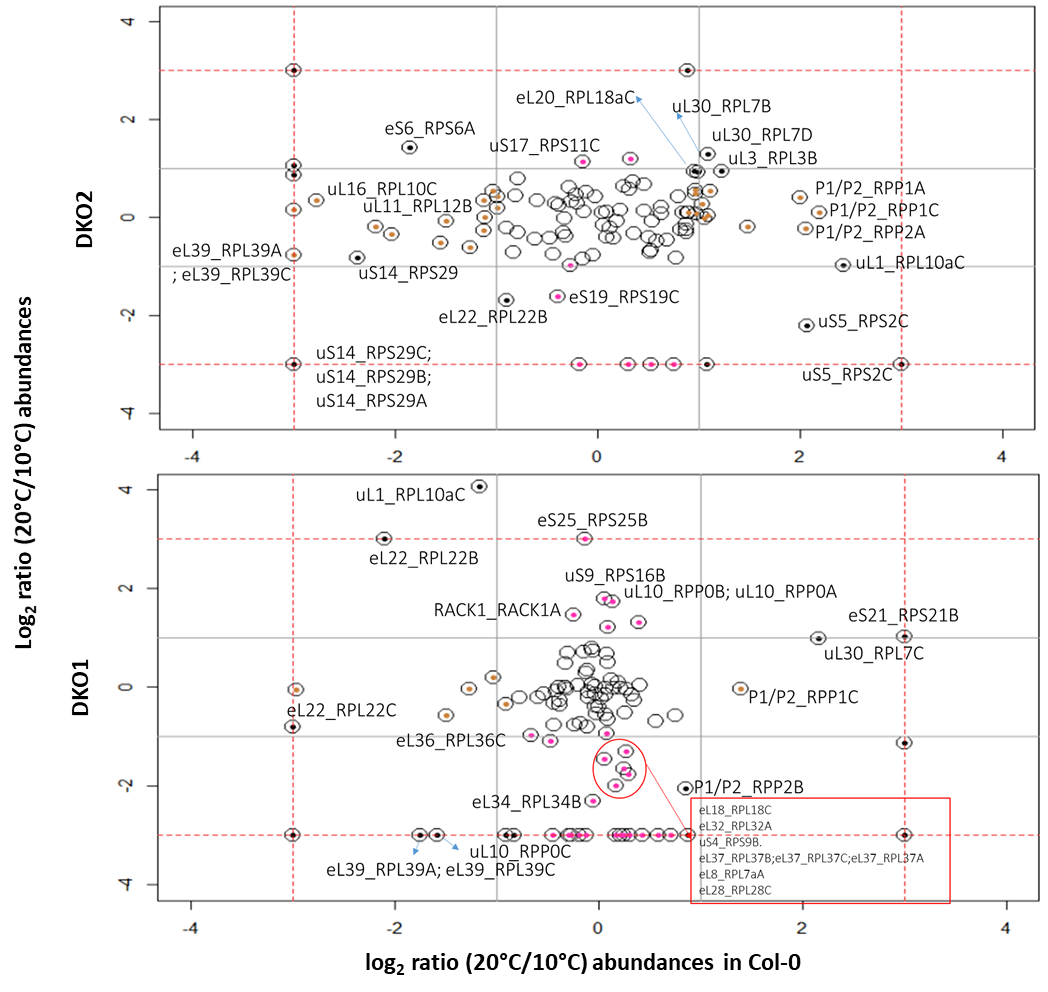

Supplement: Supplementary file 1 [file ijms-22-06160-s001.zip › Figure S5.tif]
